# Malpraxis

**Published:** 1898-07-09

**Authors:** 


### MALPRAXIS.

When an action for malpractice is brought against a medical man, everyone is apt to turn against the unfortunate defendant. For, indeed, looking back after the event, and with all the facts of the case before one, it is often pretty obvious that the treatment was not absolutely the best that could have been adopted. The doctor's defence is, of course, that he exercised due diligence and used ordinary skill, and it is a good defence so far as it goes; but, unfortunately, there is no fixed criterion of either diligence or skill by means of which a medical man can prove that he has acted rightly.

The rapid extension of specialism, and the wide publication of the details of special methods of diagnosis have a good deal to answer for. By the use of the Röntgen rays, for example, it is easy to demonstrate to the densest jury that a fracture has not been set exactly straight, or that a bit of broken needle has been left behind when it had been thought to have been entirely removed, and if it can be shown that, had the doctor but known what was wrong, his treatment would have been different, an indignant barrister may put it to the jury that means were at hand by which the defendant could have ascertained what was the matter with his patient, but he neglected to avail himself of these means, and, as a result, the plaintiff is left a cripple for life," and although the judge may point out that a Röntgen ray apparatus is not one of the things that an ordinary medical man is expected to possess, and that the defendant, being in the position of a general practitioner, was only bound to exercise ordinary care, it is by no means easy to remove from the minds of a jury the impression that the doctor ought to have made use of a means which, as they would say, is in every shop window. The same in regard to the popularisation of details of treatment. Take the question of antiseptics. A surgeon has treated a trifling wound which has been followed by septic mischief. Counsel may take a little handbook on antiseptic surgery, or on surgical technique; he may cross-examine on every detail in regard to sterilisation of the instrument, of the wound, of the surrounding skin, of the doctor's hands, and also in regard to drainage, dressings, fixation, &c., and he is sure to find something that has not been done. He may then turn in a fine frenzy to the jury and say that "the plaintiff went to this qualified medical man expecting to be treated with, and prepared to pay for, proper surgical skill, and yet he did not receive the same attention which a common tramp would have received at the hands of a junior dresser at some of the great hospitals!" Moreover, by reference to his handbooks, or, if he is driven to it, by putting surgical authorities in the witness box he may be able to prove that such like antiseptic methods are the very groundwork of modern surgery, and the ruling of the judge that gross negligence must be proved may seem to the jury to be quite beside the mark when it is shown that a fully qualified surgeon left things undone which a half-fledged dresser is taught as the very groundwork of his professional work. The matter is a very serious one, for the public will not recognise that the doctor's first duty is to exercise his discretion rather than to follow rules.

The rapid changes in modes of treatment are also a great pitfall to the practitioner. A case was tried only a little time ago in which in treating a wound of the forearm the surgeon "neglected" to suture a divided nerve, a proceeding which would have been ridiculed when half the medical men now in practice received their medical training. A case, again, was recently tried in America, in which every step in the performance of a complicated gynæcological operation was brought under review, and authorities were quoted to show that each step should have been performed differently; and what can be easier than to find authorities against every conceivable gynæcological proceeding? At the present time it is probable that an ingenious attorney and an eloquent barrister could make it very awkward for the doctor in the great majority of cases in which things go wrong in midwifery practice. Books on antiseptics might be brought forward in which the most absolute rules are laid down, and according to which a case of negligence could be made out against half the doctors in the country. Of course such charges could be refuted by good evidence of good men, showing that the definite rules laid down in the little books are not to be followed blindly, and that the qualified man's first duty is to exercise his own discretion, and use his own knowledge to the best of his power, for the benefit of his patient. But juries are stubborn things; they are all too apt to believe that doctors "hold together," and to put more faith in what is printed in a book than in what a doctor swears, as they would say, "to help his friend!"

Thus it happens that an action for malpraxis is no trifle, however correct a man's treatment may have been, and however much care he may have lavished over his case.

